# End-Stage Renal Disease Patients Undergoing Hemodialysis Have Higher Possibility of Return of Spontaneous Circulation during Out-of-Hospital Cardiac Arrest and Non-Inferior Short-Term Survival

**DOI:** 10.3390/jcm11216582

**Published:** 2022-11-06

**Authors:** Ming-Shun Hsieh, Amrita Chattopadhyay, Tzu-Pin Lu, Shu-Hui Liao, Chia-Ming Chang, Yi-Chen Lee, Wei-En Lo, Jia-Jun Wu, Vivian Chia-Rong Hsieh, Sung-Yuan Hu, Chorng-Kuang How

**Affiliations:** 1 Department of Emergency Medicine, Taipei Veterans General Hospital, Taoyuan Branch, Taoyuan 330, Taiwan; 2 Department of Emergency Medicine, Taipei Veterans General Hospital, Taipei 11217, Taiwan; 3 School of Medicine, National Yang Ming Chiao Tung University, Taipei 112, Taiwan; 4 Department of Emergency Medicine, Taichung Veterans General Hospital, Taichung 40705, Taiwan; 5 Center for Translational Genomics and Regenerative Medicine, Department of Medical Research, China Medical University Hospital, Taichung 404, Taiwan; 6 Department of Public Health, National Taiwan University, Taipei 100, Taiwan; 7 Department of Pathology and Laboratory, Taipei Veterans General Hospital, Taoyuan Branch, Taoyuan 330, Taiwan; 8 Institute of Occupational Medicine and Industrial Hygiene, College of Public Health, National Taiwan University, Taipei 100, Taiwan; 9 Department of Critical Care Medicine, Taipei Veterans General Hospital, Taoyuan Branch, Taoyuan 330, Taiwan; 10 Department of Health Services Administration, China Medical University, Taichung 404, Taiwan; 11 School of Medicine, Chung Shan Medical University, Taichung 40201, Taiwan; 12 Institute of Medicine, Chung Shan Medical University, Taichung 40201, Taiwan; 13 Department of Post-Baccalaureate Medicine, College of Medicine, National Chung Hsing University, Taichung 402, Taiwan

**Keywords:** acidosis, cardiopulmonary resuscitation (CPR), end-stage renal disease (ESRD), hyperkalemia, out-of-hospital cardiac arrest (OHCA), return of spontaneous circulation (ROSC)

## Abstract

End-stage renal disease (ESRD) patients on long-term hemodialysis (HD) have an elevated risk of sudden cardiac death. This study hypothesizes, for the first time, that these patients have a higher odds of return of spontaneous circulation (ROSC) and subsequent better hospital-outcomes, post out-of-hospital cardiac arrest (OHCA), as opposed to non-ESRD patients. A national database from Taiwan was utilized, in which 101,876 ESRD patients undergoing HD and propensity score-matched non-ESRD patients were used to conduct two analyses: (i) Cox-proportional-hazards-regression for OHCA incidence and (ii) logistic-regression analysis of attaining ROSC after OHCA, both for ESRD patients in comparison to non-ESRD patients. Kaplan-Meier analyses were conducted to determine the difference of survival rates after ROSC between the two cohorts. ESRD patients were found to be at a higher risk of OHCA (adjusted-HR = 2.11, 95% CI: (1.89–2.36), *p* < 0.001); however, they were at higher odds of attaining ROSC (adjusted-OR = 2.47, 95% CI: 1.90–3.21, *p* < 0.001), as opposed to non-ESRDs. Further, Kaplan-Meier analysis demonstrated ESRD patients with a better 30-day hospital survival rate than non-ESRD patients. Although ESRD patients had a higher risk of OHCA, they demonstrated higher possibility of ROSC and a better short-term hospital outcome than non-ESRDs. Chronic toxin tolerance and the training of vascular-compliance during regular HD may be possible explanations for better outcomes in ESRD patients.

## 1. Introduction

Non-traumatic out-of-hospital cardiac arrest (OHCA), which causes sudden cessation of heartbeat is a common acute and critical illness, posing a challenge that is faced regularly by emergency medical practitioners. However, in spite of a significant improvement in CPR practices, high mortality and morbidity is associated with OHCA, and the ROSC rates are quite low. OHCA imposes a major disease burden on the health care system [1,2]. The rate of prehospital ROSCs ranges between 16% in Italy, 30% in Ireland, up to 37% in Norway, with an overall 28.6% in all European countries, whereas in comparison, it is lower in Taiwan with a recorded 19.7% in rural areas and 27.7% in the Great Taipei Area [3]. On the other hand, the rate of the survival to discharge of OHCA patients who received CPR approximately ranges from 4.5 to 10.7% globally [4,5]. Furthermore, survival discharge is not equal to total recovery, and only 2 to 4% demonstrates better neurologic outcome [6,7]. With the elevated rate of willing-to-perform bystander CPR and advancement in emergency medical services (EMS), the ROSC rate has improved a lot during emergency treatments in the pre-hospital step. The past 10 years has witnessed improved hospital survival rates and neurologic outcomes due to subsequent in-hospital management, such as extracorporeal cardiopulmonary resuscitation (E-CPR) and targeted temperature management (TTM) [8,9]. Considering the guidelines laid out by the American Heart Association (AHA) and International Liaison Committee on Resuscitation (ILCOR) that highlights that better CPR quality is potentially the important factor toward improvement of outcomes of OHCA, uncovering possible factors that could have a positive impact on achieving sustained ROSC is an important research topic.

The prevalence of chronic kidney disease (CKD) in Taiwan is extremely high, with approximately 11.9% of the total population being affected [10]. Moreover, lack of early diagnosis leads to advanced and end-stage renal diseases (ESRD) [11]. ESRD puts a significant burden on the healthcare systems globally, and especially in Taiwan. The US Renal Data System indicates that Taiwan has had the highest incidence and prevalence of ESRD (~400 per million population) among all the enrolled countries since the year 2000 [12]. Statistics reveal that Taiwan’s 40,000 ESRD patients consumed 7% (approximately 26 billion New Taiwan dollars (NTD)) of the national health insurance budget for dialysis treatment in 2000, where, these ESRD in dialysis patients constitutes of only 0.17% of the total population [6]. ESRD patients on long-term hemodialysis (HD) has a high burden of cardiovascular diseases and are prone to high risk of sudden cardiac death [13]. Research working toward improving outcomes for a high risk population such as these is important.

Medical practice in the emergency room (ER) have allowed us to regularly experience many instances in which ESRD patients with OHCA have demonstrated repeated ROSCs as opposed to the general population. A possible explanation could be the low serum potassium levels and the lack of acidosis and hyperkalemia, in ESRD patients due to prolonged HD. Therefore, in this study we have utilized a national database of Taiwan with 101,876 enrolled ESRD patients and have conducted analyses for understanding the association of ESRD and the risk of OHCA. More importantly, we focused on the ROSC rates after CPR in OHCA patients with ESRD, in which we hypothesized that ESRD patients have a higher probability of attaining ROSC as opposed to patients with OHCA and no prior ESRD, based on our clinical experience. This has never been discussed before, and hardly any evidence exists in the literature to support this proposed hypothesis. Therefore, this is the first time we have attempted to address a question that no other study has.

## 2. Methods

### 2.1. Data Sources and Study Participants

From the catastrophic illness database of the national health insurance program of Taiwan (detailed description in Supplement Material File S1), a total of 167,373 de-identified ESRD patients who had received at least one hemodialysis treatment in a non-emergency condition during 2000–2010 were retrieved to conduct this cohort study. All patients with OHCA were sent to the emergency department (ED) by the ambulance of emergency medical service (EMS). None of the enrollees of OHCA patients with ESRD arrived from any clinic or were transferred from any other hospital. Frequency matching based on age, sex, and index year (defined as the calendar year of the index date) using propensity score was applied to obtain ESRD and non-ESRD study subjects in a 1:1 ratio. The index date is defined as the first day of ESRD diagnosis, which were then matched to a non-ESRD patient at a random date. Follow-up data for at least one year (2000–2011) for the ESRD and non-ESRD patients were obtained.

### 2.2. Variable Definitions

**Figure 1:**
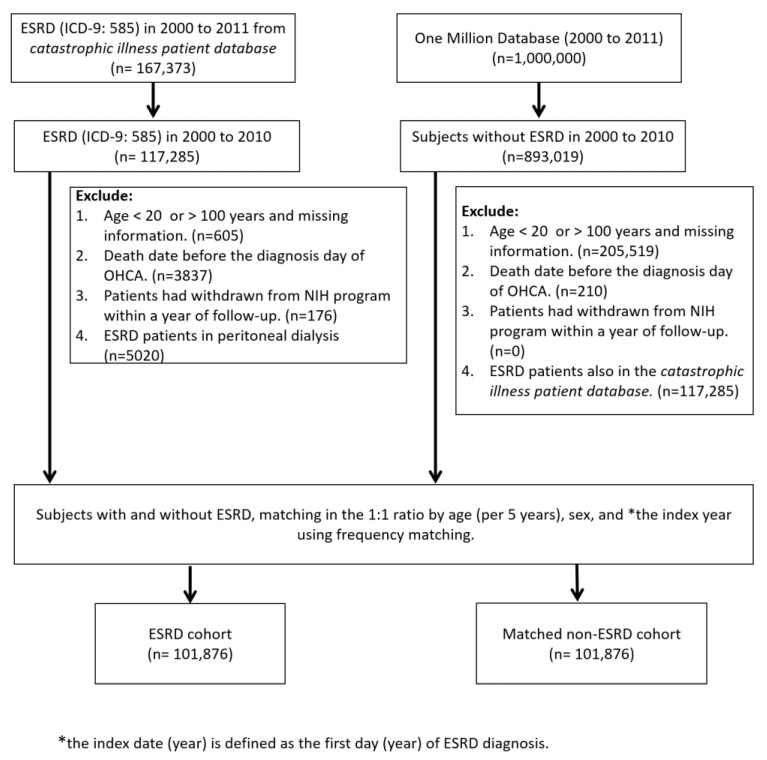
Selection algorithm for the matched ESRD in regular hemodialysis and non-ESRD patients. ESRD: End-stage renal-disease; OHCA: Out-of-hospital cardiac arrest; NIH: National Institutes of Health.

The OHCA patients who received CPR were further dichotomized into 2 groups: (1) “non-ESRD” patients who never received HD and (2) “ESRD” patients who received regular HD and were certified with catastrophic illness. The certificate exempted the ESRD patients from paying a partial fee when visiting a doctor at both the outpatient department (OPD) and ER. More importantly, these patients were waived off of a partial payment from the expensive hospitalization course. ESRD patients undergoing peritoneal dialysis (PD) were excluded from this study to avoid confusion and confounding effects due to unknown sources. The patients receiving emergent or temporary hemodialysis during the course of hospitalization were not considered as ESRD unless they were approved of the ESRD certification by the national health insurance administration. ESRD with chronic dialysis was the pre-requisite for getting enrolled into the study cohort. Patients were considered to have attained “ROSC” if they fulfilled the following criteria: (1) underwent post resuscitation by CPR (for at least 2 min), (2) received medical management, such as epinephrine or electric shock, (3) re-gained pulse and measurable blood pressure, (4) received and completed post-CPR and resuscitation care, including (portable) X-ray, complete EKG, and laboratory test data, irrespective of the number of collapses and the attainment of repeated ROSCs. Medication usage information for angiotensin-converting enzyme inhibitors (ACEIs), angiotensin receptor blockers (ARBs), aspirin, and statins were included as clinical variables in the study. All medications were considered only if they were prescribed to the patients for at least 3 months within the half year prior to the index date of the OHCA events. Socioeconomic status is highly associated with the outcome of ESRD patients [14]. The insurance premium per year for the national health insurance program was utilized as a surrogate for linking household income level for each study subject [15]. The selection algorithm for participant selection for the study cohort (ESRD patients) and the comparison (non-ESRD) cohorts are demonstrated in Figure 1.

As the NHIRD contains de-identified secondary data for research, this study was exempted from the requirement for informed consent from participants. This study was approved by the institutional review board of China Medical University (IRB# CMUH104-REC2-115) and Taichung Veterans General Hospital (IRB# CE19152A).

### 2.3. Statistical Analyses

Demographic characteristics, baseline comorbidities, medications, household incomes, and treating hospital levels for OHCA were collected for all study subjects. Continuous variables were reported as mean ± standard deviation (SD), while categorical variables were described as the number and percentage of total subjects. Differences in demographic characteristics, baseline comorbidities, medications, treating hospitals, and 28-day hospital survival rate between ESRD and non-ESRD patients were examined using the chi-squared test (categorical variables) and two-sample *t*-test (continuous variables).

Cox-proportional hazards analysis was conducted with time to incidence of OHCA as the event for patients with ESRD. Crude hazard ratios (HR) and multivariate adjusted HR were reported with 95% confidence intervals (95% CIs) to study the risk effect of ESRD toward cardiovascular events as opposed to non-ESRD patients. Next, logistic regression analysis was conducted to check the impact of ESRD on ROSC rate and 28-day hospital survival rate, for patients who suffered OHCA. Odds ratio (OR) with 95% confidence intervals (95% CIs) were calculated for each of the variables under study. Multivariate adjusted ORs were further obtained after adjusting for possible confounding effects due to age, sex, baseline comorbidities, medications, household incomes, and treating hospital levels.

Finally, Kaplan-Meier survival analysis with a two-tailed log-rank test was conducted to see the impact of ESRD on (i) OHCA incidence and (2) 28-day hospital outcome of OHCA patients who attained ROSC. A *p*-value threshold of 0.05 was used to define statistical significance. All analyses were conducted using R [16] where Cox proportional hazards regression and Kaplan-Meier analysis were conducted using the Survival package.

## 3. Results

### 3.1. Study and Comparison Cohorts

A total of 101,876 Taiwanese adults with ESRD undergoing hemodialysis were enrolled in this study between 1 January 2000 and 31 December 2020. A comparison cohort of non-ESRD patients were collected at the same time. After propensity score matching by age and sex, non-ESRD patients were retrieved in a 1:1 ratio. There was a mean follow-up period of 3.71 and 5.65 years for the ESRD and non-ESRD cohorts, respectively.

The mean age of the ESRD and non-ESRD cohort was 63.14 ± 14.29 and 62.52 ± 13.93 years, respectively. Baseline comorbidities, including hypertension (HTN), diabetes mellitus (DM), coronary artery disease (CAD), congestive heart failure (CHF), stroke, chronic obstructive pulmonary disease (COPD), and cancer were more prevalent in the ESRD cohorts. Therefore, more medications were prescribed in the ESRD cohorts including, angiotensin-converting enzyme inhibitors (ACEIs), angiotensin receptor blockers (ARBs), aspirin, and statins (Table 1).

### 3.2. Incidence Rate of OHCA in ESRD and Non-ESRD Patients

A total of 2563 ESRD and 1125 non-ESRD patients experienced OHCA, respectively, during the follow-up period. The rates of incidence were 6.78 and 1.95 person-years for each of the ESRD and non-ESRD groups, respectively. Cox regression analysis demonstrated ESRD as a significant risk factor for OHC event (crude HR = 3.42, 95% CI = 3.19–3.67, *p* < 0.001) as opposed to non-ESRD patients. After adjusting for potential confounding factors, including, age, sex, baseline comorbidities, medication, and household incomes, the ESRD continued to assert a significant risk for OHCA incidence (adjusted HR = 2.11, 95% CI = 1.89–2.36, *p* < 0.001) (Table 2).

### 3.3. Incidence and Variables Associated with ROSC

A total of 1125 patients with no prior ESRD (1.09%) had an OHCA event during the study period out of which 202 patients (17.95%) attained ROSC. On the other hand, a total of 2563 (2.52%) patients with prior ESRD had an OHCA event with a rate of attaining ROSC reported to be 42.33%. The chi-squared test demonstrated a significantly higher ROSC rate in the ESRD patients who experienced OHCA (*p* < 0.001) (Supplementary Table S1).

Logistic regression analyses demonstrated ESRD to be significantly associated with ROSC (crude OR = 3.25. 95% CI = 2.83–3.98, *p* < 0.001 and adjusted OR = 2.47. 95% CI = 1.90–3.21, *p* < 0.001). (Table 3) Statins use also showed a positive predictor for ROSC, adjusted OR = 1.46, 95% CI = 1.21–1.76, *p* = 0.001, compared with non-statin users. Also, treating OHCA patients in the medical center and regional hospital both showed higher ROSC rate with the adjusted OR = 2.44 (95% CI = 1.93–3.09, *p* < 0.001) and adjusted OR = 2.00 (95% CI = 1.60–2.5, *p* < 0.001) when comparing with treating OHCA in the district hospital.

### 3.4. Survival Analysis

**Figure 2:**
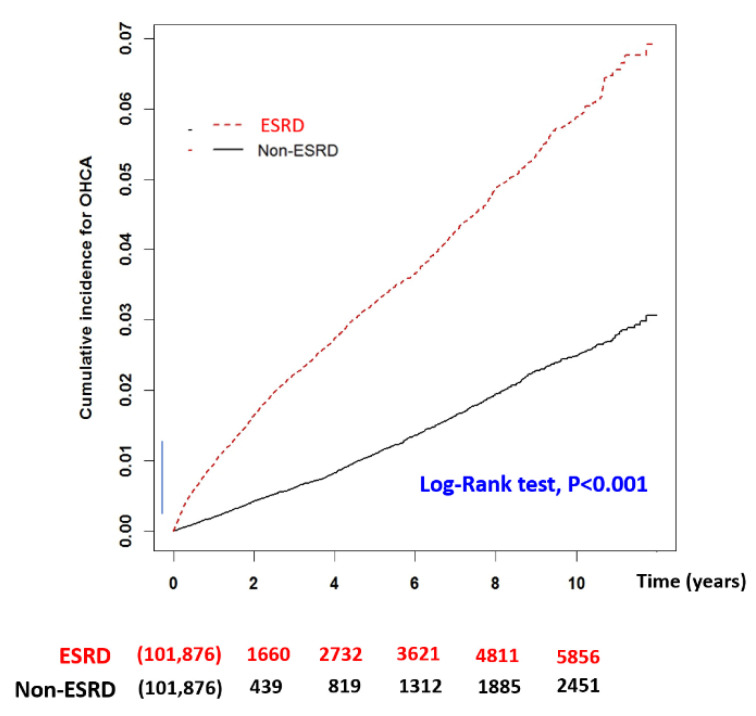
Kaplan-Meier with log-rank test to measure the cumulative incidence of OHCA between the matched ESRD in regular dialysis and non-ESRD patients. *X*-axis displays the time in years. *Y*-axis displays the cumulative incidence of OHCA. *p* < 0.05 is the threshold for significance. ESRD: End-stage-renal-disease; OHCA: out-of-hospital cardiac arrest.

**Figure 3:**
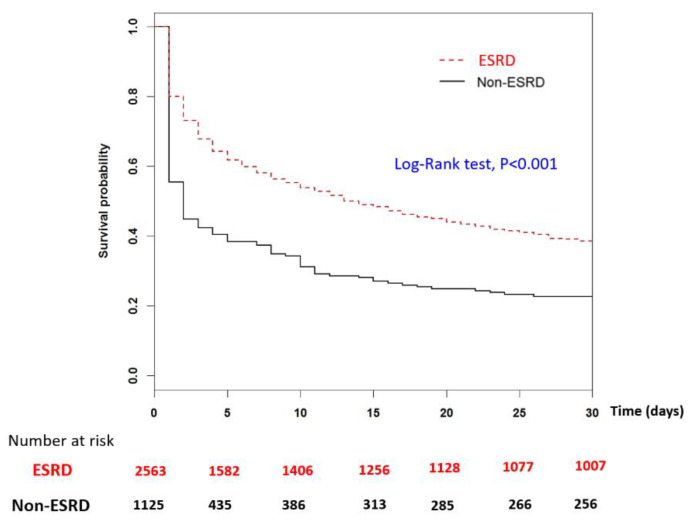
Kaplan-Meier analysis with log-rank test to measure the 30-day hospital survival rate post ROSC between the matched ESRD in regular dialysis and non-ESRD patients. *X*-axis displays the time in days. *Y*-axis displays the survival rate of admitted ROSC patients. *p* < 0.05 is the threshold for significance. ESRD: End-stage-renal-disease; ROSC: return of spontaneous circulation.

Figure 2 provides Kaplan-Meier cumulative incidence curves of OHCA event in the matched ESRD and non-ESRD groups for the follow up period. ESRD patients demonstrated a much higher incidence rate of OHCA events with a significant log-rank test *p*-value (*p* < 0.001). Figure 3 displays the Kaplan-Meier survival curves where ESRD patients demonstrated better rates of short-term survival rate (i.e., 30-days and 60-days follow up period) compared to the non-ESRD OHCA patients (log-tank test, *p* < 0.001) (Figure 3). However, for a long-term follow-up period of more than 1 year, the survival trend reversed with a better survival rate of non-ESRD patients (log-rank test, *p* < 0.001).

## 4. Discussion

A 10-year nation-wide database of patients from Taiwan were utilized in this study to demonstrate that patients with prior ESRD has significantly higher risk of OHCA, in comparison to patients with no prior ESRD, therefore reiterating the well-established hypothesis of ESRD patients having a higher risk of mortality due to cardiovascular events. Furthermore, the study presented unprecedented evidence by demonstrating that ESRD patients suffering OHCA have higher odds of attaining ROSC as compared to the non-ESRD patients. To the best of our knowledge, this study is the first to demonstrate results supporting such a hypothesis based on a large nationwide cohort. With the advancement of strategies of neuroprotection after successful resuscitation in order to reduce brain damage during OHCA, renal transplantation techniques for ESRD, and increasing prevalence of by-stander CPR, findings from this study successfully provides new insights into the ESRD patients who are on regular dialysis, irrespective of the quality of emergency services, physicians, and intensivist or nephrologist doctors. Based on the evidence from this study, it is suggested to not to consider the futility early in ESRD on HD patients due to underlying disease.

Studies have reported occurrence of significant hyperkalemia in patients during OHCA and close-chested CPR, which may lead to acidosis [17]. As hyperkalemia is commonly observed in ESRD patients, HD provides a definitive treatment for it [18]. Other studies have shown that hypokalemia had a significant association with good neurological outcome in OHCA patients leading to survival discharge [19]. This may possibly be the explanation why ESRD patients on HD demonstrates a better chance of survival by attaining ROSC through CPR. Another ongoing study by us demonstrates that ESRD patients of OHCA has lower potassium level and more severe acidosis. This could be a second reasonable explanation for better outcomes of ESRD patients. Moreover, training or improvement in vascular compliance occurs during regular hemodialysis, probably leading to better outcomes. Furthermore, ESRD patients are prescribed medications to treat hypertension, diabetes, and hyperlipidemia. These medications, such as statins, ARBs, and aspirin may provide another protective effect on the ESRD patients of OHCA. Statin therapy has been established to confer reduced risk of mortality due to cardiovascular events in ESRD patients [20]. However due to the limitation of the data at hand, we were unable to provide comprehensive proof to support this hypotheses. Further investigations are required to investigate the above-mentioned hypotheses in detail as a lot of ESRD patients undergo HD since their mid-life which may potentially provide them protective effects towards better outcomes during CPR post OHCA.

### Strengths and Limitations

One of the limitations of this study is the lack of consideration of the pre-hospital management, such as bystander CPR and telecommunicated CPR. Pre-hospital management is one of the key factors that has an effect on the final outcome of the OHCA patients. However, because all the emergency medical system personnel conduct CPR to rescue the OHCA patients according to the same nationwide regulations, we focused on the in-hospital managements in this study. Secondly, full detailed laboratory data was unavailable. This was because every hospital and physician had different strategies to manage the OHCA patients. For instance, some emergency physicians performed advanced cardiac life support (ACLS) in which they try to obtain the blood samples and laboratory report first to help infer the potential etiology of OHCA or understand the current condition of patients, while other emergency physicians may focus more on the CPR, including defibrillation, and may administer empirical medications first and upon attainment of ROSC, would initiate blood tests and imaging. This happens especially at the primary care level of hospital due to lack of resuscitation resources and staffs. Another limitation was that the ESRD patients who did not receive HD were not considered at all. This was because the primary aim was to understand how ROSC rates differed between ESRD patients with HD in comparison to non-ESRD patients, which was hypothesized based on the idea that chronic toxin tolerance and the training of vascular-compliance during regular HD allows the ESRD patients to attain ROSC with higher odds in comparison to patients without ESRD. A third limitation was that the national database used in the current study does not have information on CKD staging that could be used to classify patients according to their kidney function. This prevented us from obtaining the status of remnant kidney function in all CKD and ESRD patients and hence a detailed study of the effect of various stages of CKD and ESRD patients on the rates of attaining ROSC, could not be conducted.

The strength of this study was to provide a comprehensive study on ESRD cohorts undergoing sudden cardiac arrest, that is, OHCA and post CPR. With the advancement of kidney transplantation practices, ESRD patients in regular dialysis may have a much higher probability of ROSC that could potentially be followed by a return to a normal life without dialysis. Also, with the continuous advancement in neuroprotective strategies for reducing brain cell damage during OHCA, such as the target temperature management (TTM), novel approaches of OHCA resuscitation are coming into play. This study provides new insights into possibilities of ROSC for OHCA patients with prior ESRD based on which it is suggested to disregard the futility of extended CPRs on ESRD patients as the efforts to revive them will not be wasted due to the proven higher rates of ROSC through this study.

## 5. Conclusions

Although ESRD patients undergoing regular HD had a higher incidence rate of OHCA compared with non-ESRD patients, they demonstrated a higher possibility to attain ROSC and had a better short-term hospital outcome. This observation may be explained by the training of vascular compliance during regular hemodialysis and chronic toxin tolerance. Therefore, it is of worth to prolong CPR and spend more time resuscitating the ESRD population, especially the younger and middle-aged groups.

## Figures and Tables

**Table 1:** Demographic characteristics, baseline comorbidities, medications, household income, and treating hospital levels for OHCA between the frequencies matched ESRD and non-ESRD patients.

Variable	Non-ESRD		ESRD		*p*-Value
	(N = 101,876)		(N = 101,876)		
	n	%	n	%	
Sex					0.99
Female	51,226	50.28	51,226	50.28	
Male	50,650	49.72	50,650	49.72	
Age, years (SD)					0.99
20–29	1892	1.86	1892	1.86	
30–39	4838	4.75	4838	4.75	
40–49	12,354	12.13	12,354	12.13	
50–59	20,922	20.54	20,922	20.54	
≥60	61,870	60.73	61,870	60.73	
Mean ± SD	62.52 (13.93)		63.14 (14.29)		
Baseline comorbidity					
HTN	49,536	48.62	94,449	92.71	<0.001 ***
DM	23,762	23.32	61,365	60.23	<0.001 ***
CAD	24,438	23.99	52,132	51.17	<0.001 ***
CHF	6285	6.17	35,283	34.63	<0.001 ***
Stroke	19,297	18.94	36,226	35.56	<0.001 ***
COPD	21,171	20.78	27,135	26.64	<0.001 ***
Malignancy	3600	3.53	7108	6.98	<0.001 ***
Medications					
ACEI	11,621	11.41	46,811	45.95	<0.001 ***
ARB	10,922	10.72	49,883	48.96	<0.001 ***
Aspirin	13,684	13.43	38,707	37.99	<0.001 ***
Statin	8927	8.76	28,574	28.05	<0.001 ***
Insurance premium (NT dollars)					
<20,000	75,034	73.65	68,585	67.32	<0.001 ***
20,000–40,000	16,487	16.18	28,938	28.41	<0.001 ***
40,001–60,000	8094	7.94	3101	3.04	<0.001 ***
>60,000	2261	2.22	1252	1.23	<0.001 ***
Hospital level (for OHCA)					
Medical center	205	24.15	725	33.04	<0.001 ***
Regional hospital	431	50.77	1006	45.85	<0.001 ***
District hospital	210	24.73	463	21.10	<0.001 ***
Follow-up period (mean, median)	5.65 (5.35)		3.71 (2.91)		

*** *p* < 0.001. ESRD: End-stage renal disease; OHCA: out-of-hospital cardiac arrest. NT dollars: New Taiwan dollars

**Table 2:** The Cox regression model to measure the crude and adjusted hazard ratio and 95% confidence intervals of incidence of OHCA between the ESRD and non-ESRD patients.

Characteristics	OHCA	Crude			Adjusted		
	(n = 3688)	HR	(95% CI)	*p*-Value	aHR	(95% CI)	*p*-Value
ESRD							
No	1125	1.00	reference		1.00	reference	
Yes	2563	3.42	(3.19–3.67)	<0.001 ***	2.11	(1.89–2.36)	<0.001 ***
Sex							
Female	1832	1.00	reference		1.00	reference	
Male	1856	1.05	(0.99–1.12)	0.111	1.04	(0.96–1.12)	0.331
Age, years							
20–29	21	1.00	reference		1.00	reference	
30–39	59	1.08	(0.66–1.78)	0.759	0.79	(0.46–1.34)	0.377
40–49	256	1.94	(1.25–3.03)	0.003 **	0.92	(0.59–1.45)	0.719
50–59	682	3.54	(2.29–5.47)	<0.001 ***	0.99	(0.63–1.53)	0.947
≥60	2670	5.54	(3.6–8.51)	<0.001 ***	1.22	(0.79–1.89)	0.365
Baseline comorbidity							
HTN	3254	4.28	(3.87–4.73)	<0.001 ***	0.89	(0.78–1.02)	0.086
DM	2300	3.36	(3.14–3.59)	<0.001 ***	1.14	(1.04–1.24)	0.004 **
CAD	2027	2.82	(2.64–3.01)	<0.001 ***	1.09	(1.00–1.18)	0.040 *
CHF	1354	3.63	(3.4–3.89)	<0.001 ***	1.12	(1.03–1.22)	0.007 **
Stroke	1499	2.58	(2.41–2.76)	<0.001 ***	1.19	(1.10–1.29)	<0.001 ***
COPD	1092	1.75	(1.63–1.87)	<0.001***	1.08	(1.00–1.17)	0.061
Malignancy	171	1.27	(1.09–1.48)	0.002 **	1.22	(1.03–1.45)	0.023 *
Medications							
ACEI	1668	2.49	(2.33–2.65)	<0.001 ***	0.89	(0.82–0.96)	0.003 **
ARB	1469	2.29	(2.15–2.45)	<0.001 ***	1.25	(1.15–1.36)	<0.001 ***
Aspirin	1456	2.29	(2.14–2.45)	<0.001 ***	1.03	(0.93–1.13)	0.597
Statin	966	2.16	(2.01–2.33)	<0.001 ***	0.88	(0.83–0.97)	0.003 **
Insurance premium (NT dollars)							
<20,000	2958	1.00	reference		1.00	reference	
20,000–40,000	602	0.61	(0.56–0.66)	<0.001 ***	0.88	(0.79–0.97)	0.012 *
40,001–60,000	103	0.38	(0.31–0.46)	<0.001 ***	0.98	(0.78–1.22)	0.831
>60,000	25	0.30	(0.20–0.45)	<0.001 ***	0.76	(0.49–1.18)	0.223

Adjusted HR: adjusted for ESRD, age, sex, comorbidities, medications, and insurance premium in the Cox regression model. * *p* < 0.05, ** *p* < 0.01, *** *p* < 0.001. ESRD: End-stage renal disease; OHCA: out-of-hospital cardiac arrest. NT dollars: New Taiwan dollars.

**Table 3:** Logistic regression model measured odds ratio with 95% confidence interval of ROSC associated with ESRD undergoing hemodialysis and other variables in OHCA patients.

Characteristics	Crude			Adjusted		
	OR	(95% CI)	*p*-Value	OR	(95% CI)	*p*-Value
ESRD						
No	1.00	reference		1.00	reference	
Yes	3.25	(2.83–3.98)	<0.001 ***	2.47	(1.90–3.21)	<0.001 ***
Gender						
Female	1.00	reference		1.00	reference	
Male	0.80	(0.70–0.92)	0.001 **	0.85	(0.73–1.00)	0.050
Age, years (SD)						
20–29 years	1.00	reference		1.00	reference	
30–39 years	0.81	(0.30–2.20)	0.677	0.80	(0.26–2.40)	0.690
40–49 years	0.93	(0.38–2.26)	0.865	1.01	(0.40–2.57)	0.980
50–59 years	0.75	(0.32–1.80)	0.521	0.85	(0.35–2.1)	0.730
≥60 years	0.52	(0.22–1.23)	0.137	0.71	(0.29–1.73)	0.450
Baseline comorbidity						
(ref = non-)						
HTN	1.76	(1.40–2.22)	<0.001 ***	0.91	(0.67–1.23)	0.521
DM	1.33	(1.15–1.53)	<0.001 ***	0.80	(0.66–0.97)	0.023
CAD	1.20	(1.05–1.38)	0.009 **	1.00	(0.83–1.20)	0.988
CHF	1.27	(1.10–1.46)	<0.001 ***	1.04	(0.87–1.24)	0.687
Stroke	0.93	(0.81–1.07)	0.321	1.02	(0.86–1.21)	0.849
COPD	0.91	(0.78–1.05)	0.196	1.07	(0.89–1.29)	0.456
Malignancy	0.74	(0.53–1.04)	0.080	0.89	(0.61–1.31)	0.558
Drug use						
ACEIs	1.12	(0.98–1.28)	0.111	0.95	(0.8–1.13)	0.591
ARBs	1.59	(1.39–1.83)	<0.001 ***	1.22	(1.02–1.45)	0.026 *
Statins	2.39	(2.08–2.75)	<0.001 ***	1.46	(1.21–1.76)	<0.001 ***
Aspirin	1.09	(0.95–1.25)	0.232	0.86	(0.72–1.03)	0.095
Insurance premium (NT dollars)						
<20,000	1.00	reference		1.00	reference	
20,000–40,000	1.66	(1.39–1.99)	<0.001 ***	1.26	(1.01–1.56)	0.039
40,001–60,000	0.96	(0.63–1.46)	0.839	0.80	(0.49–1.34)	0.399
>60,000	0.79	(0.33–1.90)	0.597	0.41	(0.13–1.26)	0.118
Hospital level						
Medical center	2.60	(2.07–3.26)	<0.001 ***	2.44	(1.93–3.09)	<0.001 ***
Regional hospital	1.99	(1.61–2.47)	<0.001 ***	2.00	(1.60–2.5)	<0.001 ***
District hospital	1.00	reference		1.00	reference	

* *p* < 0.05, ** *p* < 0.01, *** *p* < 0.001. Abbreviation: OR, odds ratio; CI, confidence interval; ESRD: end-stage renal disease; ROSC: return of spontaneous circulation; OHCA: out-of-hospital cardiac arrest. Adjusted OR: adjusted for ESRD, age, gender, baseline comorbidity, drug use, insurance premium, and hospital level in logistic regression model. NT dollars: New Taiwan dollars.

## Data Availability

The data presented in this study are available on request from the corresponding author. The data are not publicly available due to Taichung Veterans General Hospital privacy regulations.

## Supplementary Materials

The following supporting information can be downloaded at: https://www.mdpi.com/article/10.3390/jcm11216582/s1. Supplemental Material File S1. Table S1: Comparison of incidence rate of ROSC in ESRD and non-ESRD patients with OHCA.
