# Supplementary material for: End-Stage Renal Disease Patients Undergoing Hemodialysis Have Higher Possibility of Return of Spontaneous Circulation during Out-of-Hospital Cardiac Arrest and Non-Inferior Short-Term Survival
Source: J Clin Med. 2022 Nov 6;11(21):6582. doi: 10.3390/jcm11216582 (PMC9659049; doi:10.3390/jcm11216582)
Supplement: Supplementary file 1 [file jcm-11-06582-s001.zip › jcm-1970194-supplementary.pdf]

Supplementary data

Ming-Shun Hsieh [1,2,3,4], Amrita Chattopadhyay [5], Tzu-Pin Lu [6], Shu-Hui Liao [7], Chia-Ming Chang [2,3,8], Yi-Chen Lee [1], Wei-En Lo [1], Jia-Jun Wu [9], Vivian Chia-Rong Hsieh [10], Sung-Yuan Hu [4,11,12,13, *,†] and Chorng-Kuang How [2,3,*,†]

[1] Department of Emergency Medicine, Taipei Veterans General Hospital, Taoyuan Branch, Taoyuan 330, Taiwan
[2] Department of Emergency Medicine, Taipei Veterans General Hospital, Taipei, Taiwan
[3] School of Medicine, National Yang Ming Chiao Tung University, Taipei 112, Taiwan
[4] Department of Emergency Medicine, Taichung Veterans General Hospital, Taichung 40705, Taiwan
[5] Center for Translational Genomics and Regenerative Medicine, Department of Medical Research, China Medical University Hospital, Taichung 404, Taiwan
[6] Department of Public Health, National Taiwan University, Taipei 100, Taiwan
[7] Department of Pathology and Laboratory, Taipei Veterans General Hospital, Taoyuan Branch, Taoyuan 330, Taiwan
[8] Institute of Occupational Medicine and Industrial Hygiene, College of Public Health, National Taiwan University, Taipei 100, Taiwan
[9] Department of Critical Care Medicine, Taipei Veterans General Hospital, Taoyuan Branch, Taoyuan 330, Taiwan
[10] Department of Health Services Administration, China Medical University, Taichung 404, Taiwan
[11] School of Medicine, Chung Shan Medical University, Taichung 40201, Taiwan
[12] Institute of Medicine, Chung Shan Medical University, Taichung 40201, Taiwan
[13] Department of Post-Baccalaureate Medicine, College of Medicine, National Chung Hsing University, Taichung 402, Taiwan
* Correspondence: ckhow@vghtpe.gov.tw; song9168@pie.com.tw
† The authors contributed equally to this work.

Supplemental Material File S1

We conducted this cohort study using the national database between 2000 and 2011 from the National Health Insurance Research Database (NHIRD) of Taiwan. The National Health Insurance Program was launched by the National Health Insurance Administration (NHIA) in 1995 with over 99% of Taiwan residents (>23.03 million residents) participating in the government-run single-payer National Health Insurance (NHI) program. The NHIA releases deidentified patient information and claims data to the National Health Research Institute (NHRI) for the NHIRD building. The confidentiality and credibility of these data are strictly maintained in accordance with the NHIRD regulations, with documented high quality in previous studies.

The database in the NHIRD contains de-identified basic demographic information, disease diagnoses, prescriptions, procedures, and examinations for each enrollee in hospitalization or outpatient visit records before, during and after the index hospitalization. The diagnosis codes, in accordance with the International Classification of Diseases, Ninth Revision, Clinical Modification (ICD-9-CM), is used throughout this study. Baseline comorbidities were defined using the ICD-9-CM. If a patient was defined to have one baseline comorbidity, such as COPD, they needed to have at least one of any the records of that (1) ≥2 outpatient visits for the same main diagnosis or (2) one specific hospitalization diagnosis record for the specific disease. The diagnoses made within a 1-year period before the index date were considered the underlying comorbidities of a patient.

Table S1. Comparison of incidence rate of ROSC in ESRD and non-ESRD patients with OHCA.

Variable	non-ROSC	ROSC	Total	P-value
	n	n		
Non-ESRD + OHCA (n = 1125)	923 (82.04%)	202 (17.96%)	1125	<0.001***
ESRD + OHCA (*n* = 2563)	1478 (57.67%)	1085 (42.33%)	2563	

***P<0.001; ESRD: End-stage-renal-disease; ROSC: return of spontaneous circulation; OHCA: Out of hospital cardiac arrest.
